# Evaluation of initial setup errors of two immobilization devices for lung stereotactic body radiation therapy (SBRT)

**DOI:** 10.1002/acm2.12093

**Published:** 2017-05-14

**Authors:** Yoshihiro Ueda, Teruki Teshima, Higinia Cárdenes, Indra J. Das

**Affiliations:** ^1^ Department of Radiation Oncology Indiana University School of Medicine Indianapolis IN USA; ^2^ Department of Radiation Oncology Osaka International Cancer Institute Chuo‐ku Osaka Japan; ^3^ The Arnold Center for Radiation Oncology New York Presbyterian Queens Weill Cornell Medicine New York NY USA; ^4^ Department of Radiation Oncology New York university Langone Medical Center New York NY USA

**Keywords:** immobilization device, lung, SBRT, setup accuracy, setup efficacy

## Abstract

The aim of this study was to investigate the accuracy and efficacy of two commonly used commercial immobilization systems for stereotactic body radiation therapy (SBRT) in lung cancer. This retrospective study assessed the efficacy and setup accuracy of two immobilization systems: the Elekta Body Frame (EBF) and the Civco Body Pro‐Lok (CBP) in 80 patients evenly divided for each system. A cone beam CT (CBCT) was used before each treatment fraction for setup correction in both devices. Analyzed shifts were applied for setup correction and CBCT was repeated. If a large shift (>5 mm) occurred in any direction, an additional CBCT was employed for verification after localization. The efficacy of patient setup was analyzed for 105 sessions (48 with the EBF, 57 with the CBP). Result indicates that the CBCT was repeated at the 1^st^ treatment session in 22.5% and 47.5% of the EBF and CBP cases, respectively. The systematic errors {left–right (LR), anterior–posterior (AP), cranio‐caudal (CC), and 3D vector shift: (LR ^2^ + AP ^2^ + CC ^2^)^1/2^ (mm)}, were {0.5 ± 3.7, 2.3 ± 2.5, 0.7 ± 3.5, 7.1 ± 3.1} mm and {0.4 ± 3.6, 0.7 ± 4.0, 0.0 ± 5.5, 9.2 ± 4.2} mm, and the random setup errors were {5.1, 3.0, 3.5, 3.9} mm and {4.6, 4.8, 5.4, 5.3} mm for the EBF and the CBP, respectively. The 3D vector shift was significantly larger for the CBP (*P* < 0.01). The setup time was slightly longer for the EBF (EBF: 15.1 min, CBP: 13.7 min), but the difference was not statistically significant. It is concluded that adequate accuracy in SBRT can be achieved with either system if image guidance is used. However, patient comfort could dictate the use of CBP system with slightly reduced accuracy.

## 1 INTRODUCTION

Stereotactic body radiation therapy (SBRT) or stereotactic ablative radiation therapy (SABR) is becoming a standard treatment modality for early stage inoperable small localized lesions in the lung and liver as well as for spinal lesions.^1^, ^2^, ^3^, ^4^, ^5^, ^6^, ^7^ However, the success of these therapies depends on the efficacy of the fixation device used during immobilization. Before the introduction of image‐guided radiotherapy (IGRT), the stereotactic body frames were used to minimize motion and try to keep lesion stationary with respect to the frame coordinate.^8^ The Elekta Body Frame (EBF: Elekta AB, Stockholm, Sweden) was the first device to achieve popularity.^6^ Nevertheless, the recent introduction of kilovoltage imaging in modern linear accelerators has aided the evaluation of body fixation that rendered the stereotactic body frame less important.^9^ Consequently, rigid immobilization is losing popularity due to imaging‐based SBRT processes. This has led to many other devices currently available in the market.^10^, ^11^, ^12^, ^13^, ^14^, ^15^, ^16^, ^17^

The success of SBRT depends on the accuracy of localization and ultimately on the treatment. Generally, ≤3 mm setup uncertainty and tumor motion are acceptable in a good clinical practice, although 5 mm are acceptable in many institutions.^4^, ^5^, ^6^, ^7^, ^13^ The rigid EBF device is restrictive in many respects due to its size, especially for obese and noncompliant patients, whereas a newer device, the Civco Body Pro‐Lok (CBP: CIVCO Medical Solutions, Orange City, IA, USA), is an open architect and provides wider dimensions and flexibility in setting up these types of patients. In some institutions, SBRT for lung was performed with the CBP.^11^, ^14^, ^15^

There have been great discussions regarding setup accuracy for the EBF.^6^, ^16^, ^17^, ^18^, ^19^ Wulf et al.^16^ and Guckenberger et al.^17^ found that positions of lung tumors have low reproducibility relative to external stereotactic coordinates and the bony anatomy. Foster et al.^18^ analyzed effectiveness of the EBF in various treatment sites, such as, lung, liver, prostate, and spine. Worm et al.^19^ reported inter‐ and intrafractional set up errors for the EBF in lung and liver. However, comparative data are not available regarding the accuracy and efficacy of EBF and CBP in IGRT‐based SBRT. This study attempted to fill this gap by investigating these two commonly used commercial immobilization systems with an abdominal compression plate to evaluate their efficacy in setup accuracy for SBRT.

## 2 MATERIALS AND METHODS

This retrospective study of 80 patients, which was under Institutional Review Board (IRB) exempt status, analyzed the setup errors for the EBF and the CBP, which were used for 40 patients who had received SBRT for lung cancer. The EBF patients included 20 cases of upper lobe, 3 of middle lobe, and 17 of lower lobe cancer, whereas the CBP patients included 20, 4, and 16 respective cases.

### 2.A Immobilization devices

**Figure 1:**
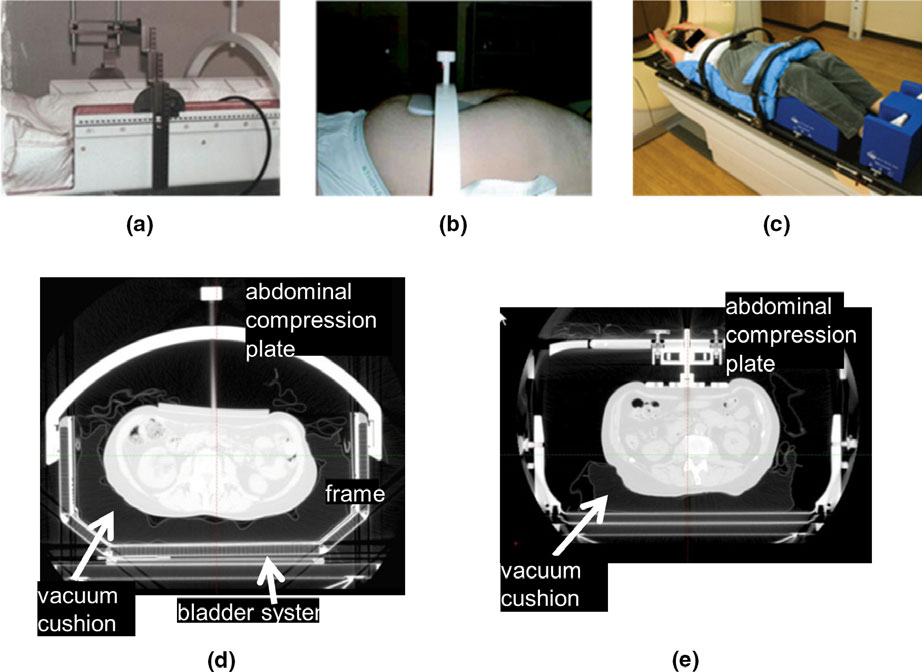
Two immobilization systems for SBRT. (a) The rigid frame and coordinate systems for the EBF; (b) The abdominal compression plate for the EBF; (c) CBP; (d) Locations of some parts for the EBF; (e) Locations of some parts for the CBP.

Fig. 1 shows the two devices used for immobilization of patients during the CT simulation and treatment with SBRT. The EBF has a rigid body frame and various components, including a custom‐made vacuum cushion, an abdominal compression plate, and a pneumatic bladder system that tilts the base of the frame without changing the patient position. The bladder system, consisting of a balloon under the frame, is mainly used for the correction of rotation by adjusting the amount of air in the balloon. The EBF system also features a stereotactic coordinate mapping system that allows localization of the isocenter from CT data. In contrast, the CBP is an open structure without a rigid frame and consists of a custom‐made vacuum cushion under the patient's body, an abdominal compression plate or belt, knee and foot supports, and a wing board (Fig. 1). Both systems have an abdominal compression plate that is used to limit the diaphragmatic excursion visualized under fluoroscopy to ≤5 mm for CT simulation.

### 2.B Treatment planning

Depending on the patient's body structure and comfort, one of the devices (EBF or CBP) was chosen at the time of simulation. However, there was an initial institutional bias based on our vast amount of experience with EBF.^1^, ^2^ Because we had a conventional simulator with fluoroscopic option (Nucletron Odelft Simulix‐HP, Elekta Medical System) adjacent to our CT scanner, patients were set up in a conventional simulator room with either of the two immobilization devices. The diaphragmatic excursion was visualized with fluoroscopic images within ≤5 mm by adjusting the abdominal pressure either by the compression plate or the belt, while maintaining the patient's comfort. Whenever necessary, oxygen was provided for comfort to patients to achieve the institutional goal of ≤5 mm diaphragmatic excursion. Following satisfactory setup, the patients were moved in the next room with the same immobilization device for four‐dimensional CT (4DCT).

The patient‐specific respiratory motion of the tumor was recorded using 4DCT on a wide‐bore CT scanner (Philips Medical Systems, Milpitas, CA, USA), equipped with a strain belt and gauge over the patient's abdomen. The internal target volume (ITV) was based on maximum intensity projection (MIP) reconstructed from the phase binning. The planning target volume (PTV) was created by adding a 5 mm expansion in the axial (left–right, LR; and anterior–posterior, AP), and 10 mm expansion in the longitudinal (superior–inferior) directions from the gross tumor volume (GTV) of the ITV.

Treatment planning was performed with an Eclipse system (Varian Medical Systems, Palo Alto, CA, USA), using the analytical anisotropic algorithm (AAA) for inhomogeneity corrections. A 6‐MV photon was typically used for the SBRT treatment. The planning process included 5–12 noncoplanar beams to deliver 24–72 Gy in three to five fractions depending on the type of protocol (external or internal). The dose prescription was typically an 80% isodose line that covered at least 95% of the PTV in three‐dimensional conformal radiotherapy (3DCRT); however, in intensity‐modulated radiotherapy (IMRT), no normalization was needed. Treatment fractions were administered once per day every 2–3 days. The noncoplanar beam arrangements were necessary for SBRT to meet the rigid criteria for adequate target coverage without exceeding normal tissue constraints, contrary to the conclusions drawn by Chang et al.,^20^ which indicated no superiority of noncoplanar fields.

### 2.C Data collection for setup accuracy

Patients were treated on a Varian Trilogy linear accelerator (Varian Medical Systems) equipped with a gantry‐mounted on‐board imager (OBI). The daily quality assessment showed that the difference between the radiation isocenter and CBCT isocenter was smaller than 1.0 mm. Daily setup within the body frame apparatus was confirmed with the aid of sternal and tibial tattoos, and the treatment isocenter was verified before each treatment with the EBF. Laser‐based alignment with detailed simulation‐based instruction ensured a fixed rotation as well as the lateral and cranio‐caudal (CC) position of the patient for both systems. We identified the AP position and the LR position using skin marks for the CBP and the frame coordinates for the EBF. Cone beam CT (CBCT) was acquired before each treatment session, for setup verification. A physician registered the tumor on the CBCT according to the tumor characteristics.

Table rotation was not performed as we do not have the 6 degree of freedom table. However, based on our earlier study indicating that rotation can be achieved by coordinate translation was used.^21^ Couch shifts associated with the interfractional setup errors were analyzed to assess the accuracy of the initial CBCT setups of each immobilization system along the three axes and by means of a 3D vector (3D vector: the root sum of LR^2^, AP^2^, and CC^2^). If a large error (>5 mm) was detected in any direction (LR, AP, CC), an additional CBCT was acquired for verification of the localization after correcting the setup errors. Couch shifts of additional CBCT were applied and also recorded to analyze residual setup errors. Positive values indicated to move couch in left, posterior, and caudal direction. Systematic and random errors constituted the mean and the standard deviation (SD) of setup errors, respectively, for each patient for all treatment sessions. The group mean values were defined as the average value of all fractions from all patients. The total SD of systematic errors (Σ) and the total SD of random errors (*σ*), very similar to the one described by Hansen et al.,^5^ were calculated for each setup method for both immobilization systems in three directions and a 3D vector calculated by the formula of (LR^2^+AP^2^+CC^2^)^1/2^. The registered shift represented the couch shift to correct set up errors and was defined as the absolute value for setup errors in each direction.

### 2.D Data collection for setup efficacy

In addition to the accuracy of patient setup attained with each immobilization system, we also analyzed the positioning and preparation times for the treatment of 41 patients (15 with the EBF; 26 with the CBP) who had undergone treatment from October 2013 to October 2014 in 105 sessions (48 with the EBF; 57 with the CBP). The positioning time, as an indication of the ease and efficacy of the system, was defined as the interval between the patient entering the treatment room and the start of the first acquisition of the CBCT. The preparation time was defined as the interval between the patient entering the treatment room and the start of treatment, not including the time spent waiting for a physician to arrive at the treatment control room.

### 2.E Statistical analysis

An unpaired independent t‐test was used to compare the data for accuracy and efficacy. A value of *P* < 0.05 was defined as statistically significant.

## 3 RESULTS

### 3.A Patient characteristics and acquisition of data for repeat CBCT

The means ± SD for the weight (kg), body mass index (BMI) kg m^−2^, and GTV (cm^3^) of the patients treated with the EBF and the CBP were 69.8 ± 15.6 and 75.2 ± 21.1 kg, 25.2 ± 5.7 and 27.0 ± 7.1 kg m^−2^, and 13.0 ± 12.6 and 13.3 ± 15.2 cm^3^, respectively. As expected, it indicates that heavier patients were used with CBP system. The rates of CBCT reacquisition after the first acquisition were 20.5% (33 sessions out of 161) and 27.8% (42 out of 151) for the EBF and CBP respectively. The maximum imaging rate was observed in the first session for both systems: for example, 22.5% for EBF and 47.5% for the CBP. For the EBF, the CBCT repeat rates were 20.0, 17.5, 18.2, and 25% for the second, third, fourth, and fifth sessions, respectively, and the corresponding rates were 32.5, 37.5, 31.8, and 22.2% for the CBP. From the first to the fourth session, the CBCT repeat rate was higher in the CBP compared to the EBF.

### 3.B Initial setup accuracy

The means ± SDs of the registered shifts for the initial setup of the two immobilization systems in the LR, AP, and CC positions were, 3.8 ± 2.6, 3.1 ± 2.3, and 3.4 ± 2.0 mm for the EBF and 3.9 ± 2.2, 4.1 ± 3.0, and 5.1 ± 3.3 mm for the CBP, respectively. The registered shift along the CC axis was larger for the CBP than for the EBF, and the difference was statistically significant. Table 1 summarizes the group means and the systematic and random localization for the initial setup errors. The systematic error along the AP axis was significantly larger for the EBF (*P* < 0.05), which showed a systematic error in the downward shift in the position of the couch along the AP direction. However, the SD was smaller for the EBF than for the CBP, while the 3D vector was significantly larger for the CBP (*P* < 0.01).

**Table 1:** Group mean values and systematic and random errors for the initial setup (mm) of the EBF and CBP immobilization systems

EBF	LR	AP	CC	3D
Group mean	0.5	2.3	0.7	7.1
Ʃ	3.7	2.5	3.5	3.1
*σ*	5.1	3.0	3.5	3.9
**CBP**	**LR**	**AP**	**CC**	**3D**
Group mean	0.4	0.7	0.0	9.2
Ʃ	3.6	4.0	5.5	4.2
*σ*	4.6	4.8	5.4	5.3

**Figure 2:**
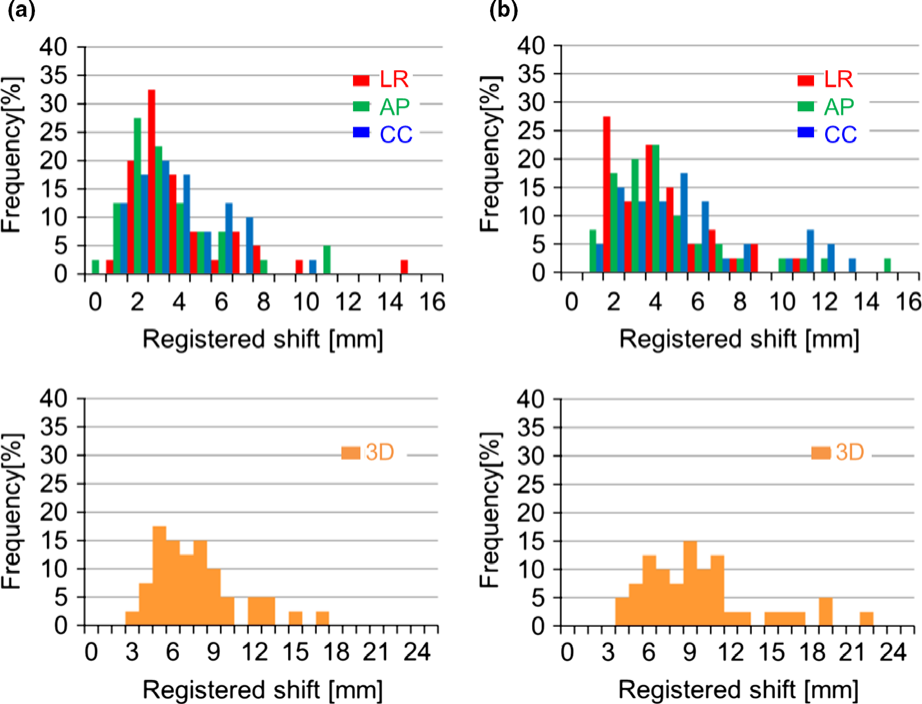
Histograms of the absolute registered shifts in relation to the initial setup for the two immobilization systems; (a) EBF and (b) CBP.

**Figure 3:**
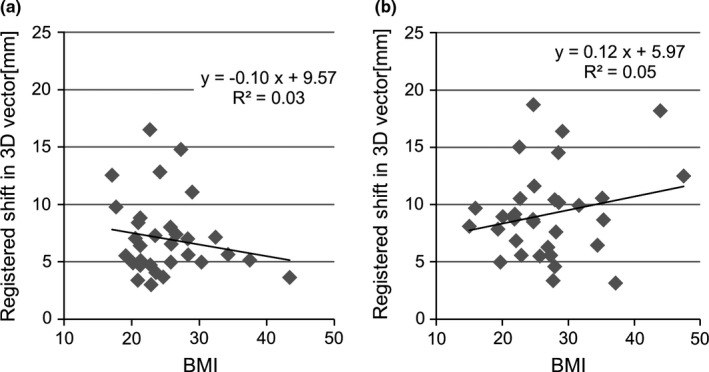
Correlations between the absolute registered shifts in relation to the initial setup for the 3D vector and the patient's body mass index (BMI) with the two immobilization systems; (a) EBF and (b) CBP.

Fig. 2 shows these four registered shift distributions. The ratio (%), {LR, AP, CC, 3D vector shift}, of the cases with registered shifts of more than 5 mm were {20.0, 12.5, 25.0, 70.0%} and {22.5, 22.5, 37.5, 87.5%} for the EBF and CBP, respectively, and for registered shifts of more than 10 mm were {2.5, 5.0, 0.0, 15%} and {2.5, 7.5, 15, 32.5%}, respectively, and for registered shifts more than 3 mm were {45.0, 35.0, 50.0, 97.5%} and {60.0, 55.0, 67.5, 100%} for the EBF and CBP, respectively. As Fig. 3 indicates, no significant correlation was indicated between the registered shifts and the BMI for the EBF and CBP.

**Figure 4:**
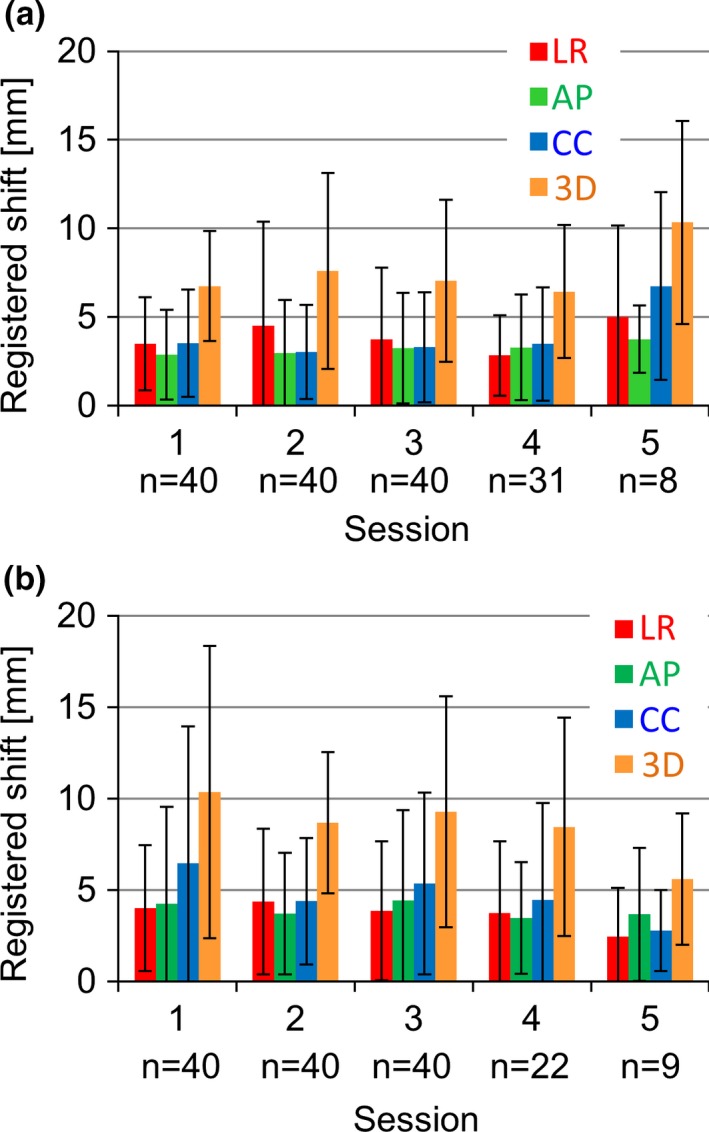
Daily changes in the initial absolute registered shift for the two immobilization systems; (a) EBF and (b) CBP.

Fig. 4 shows the daily changes in the mean and SD of initial setup shifts for the EBF and CBP. From the first to the fourth sessions, initial setup shifts were more stable for the EBF than for the CBP. The error for the 3D vector was significantly larger (*P* < 0.01) for the CBP than for the EBF. The largest 3D vector shift occurred during the first session of the CBP.

### 3.C Accuracy of setup with CBCT

The number of cases that needed the repeat CBCT were 16 and 28 cases for the EBF and CBP, respectively. The registered shifts {LR, AP, CC, and 3D vector} measured by repeated CBCT were {0.8 ± 1.0, 1.2 ± 1.3, 0.8 ± 1.0, and 1.9 ± 1.8} mm for EBF and {2.0 ± 1.9, 2.5 ± 2.0, 1.4 ± 1.1, and 4.0 ± 2.5} mm for CBP, respectively. The registered shifts for EBF were significantly smaller (*P* < 0.01) than those for the CBP in all directions. The registered shifts using repeat CBCT were smaller than with the initial setup in any direction for either immobilization system. Table 2 summarizes the group means, systematic localization errors, and random errors for the second setup with repeat CBCT. The systematic and random errors both improved when compared to the initial setup errors with both immobilization systems.

**Table 2:** Group mean values and systematic and random errors for the CBCT setup (mm) of the EBF and CBP immobilization systems

EBF	LR	AP	CC	3D
Group mean	0.0	0.0	0.0	1.9
Ʃ	1.4	1.7	1.2	1.8
*σ*	1.0	1.5	0.9	1.1
**CBP**	**LR**	**AP**	**CC**	**3D**
Group mean	0.0	1.0	1.0	4.0
Ʃ	2.5	2.9	1.4	2.5
*σ*	2..5	2.2	2.1	2.6

### 3.D Setup efficacy

Setup time was longer for the EBF (15.1 min) than for the CBP (13.7 min), whereas the preparation time was shorter for the EBF (23.5 min) than for the CBP (24.5 min). The overall efficacy, however, showed no significant difference between the two immobilization systems. A greater number of repeated CBCTs were required for the CBP than for the EBF to verify the initial setup accuracy, which was the main reason for the longer treatment preparation time for the CBP than for the EBF. The setup time could also have been affected by the training and familiarity of the staff with each system as well as by the cooperation of the patients. These are some factors that are not easy to quantify; however, we have a long history of SBRT usage^1^, ^2^ and our staff is fully trained.

## 4 DISCUSSION

This study compared the initial setup accuracy and effectiveness of two immobilization devices used for SBRT in conjunction with IGRT: the EBF with a rigid frame and the free flowing CBP without a frame. For SBRT, huge transformation occurs from stereotaxic position to image guidance where immobilization plays a significant role for time saving and accuracy. The initial setup is important to reproduce the delivered dose based on the treatment planning. Few studies have evaluated the setup efficacy of the CBP^11^ based on time and accuracy, whereas many reports have been published regarding the setup for the EBF.^6^, ^16^, ^17^, ^18^, ^19^ The various immobilization systems available for SBRT have been the subject of major discussions. Because the CBP is a new immobilization system, its setup accuracy and effectiveness need to be compared to those of traditional immobilization systems, such as the EBF.

The initial setup using the EBF in the AP direction showed a large group mean error along the posterior, couch‐down, direction in our study. In contrast, Guckenberger et al.^17^ and Foster et al.^18^ detected a group mean error for the EBF along the anterior because the patient's body had shifted downward due to small leaks in the vacuum cushion or to a relaxation effect. At our institution, a longer time at the simulation was required for patients to hold the supine position when compared to the treatment time. For this reason, the patients may have been more relaxed during the simulation to acquire 4DCT at CT simulation than at treatment. In addition, the tumors on 4DCT at CT simulation were located lower than those on CBCT at treatment. On the other hand, the CBP used skin marks on the patients for the initial setup. The CBP showed only minor changes due to the relaxation effect or to small leaks in the vacuum of the cushion, so the group mean error was small in the AP direction.

The initial registered shift in the CC direction was larger than that in any other direction for the CBP. In addition, the shift was significantly larger in comparison to the shift seen with the EBF. Gutierrez et al.^11^ found that the systematic error for the CBP was largest in the CC direction, which agrees with our results. This error was also larger than any reported for the EBF. The EBF uses two laser markers to determine the sternal and tibial positions of patients. Although the CBP offers a simple setup that uses only skin marks on a target position on the patient's chest to determine the patient's position along the CC direction, the EBF presumably shows greater accuracy than the CBP in the CC direction.

We also evaluated the initial setup errors for the tumor‐matching localization. Soft tissue registration, wherever possible, is done routinely. Since it is image guided, physician decides about the image fusion and expected shift. Worm et al.^19^ found a significant correlation between patient BMI and the mean 3D vector of the initial setup error for bone‐matching localization. No researcher assessed the correlation between BMI and initial setup error for tumor matching. If the BMI is so large, it is more likely that patient will not fit in rigid frame. Hence, we hypothesize that EBF has larger errors in proportion to BMI. Our results indicated that the accuracy of setup using tumor matching was not affected by patient habitus because the respiratory condition of the patients has a stronger effect on the tumor position.

From the first session to the fourth session, the EBF displayed a more stable setup accuracy than the CBP, which resulted from the method used for patient setup with the two immobilization systems. In this context, daily changes (e.g., a different therapist for each session) presumably affected the simple setup associated with the CBP. The setup method for the EBF, on the other hand, is generally complicated and intricate. Therefore, the EBF offers a more stable initial setup than the CBP in terms of daily changes in the accuracy of the initial setup.

The registered shifts {LR, AP, CC, and 3D vector} measured by repeated CBCT were {0.8 ± 1.0, 1.2 ± 1.3, 0.8 ± 1.0, and 1.9 ± 1.8} mm for EBF. Similar results were reported by Shah et al.,^22^who found a difference of 2.3 mm in the mean values for the initial setup errors and second setup errors for the 3D vector for the EBF. Grills et al.^23^ found the IGRT to be helpful in correcting the setup errors because its use resulted in an improvement of more than 2.0 mm in all directions for the EBF. However, there were some setup errors with additional CBCT. We found that the second registered shifts for EBF were significantly smaller than those for CBP in all directions. This may be due to the design of the systems, EBF being more rigid and CBP more free‐flow. Other conditions except the body frame were almost same between both immobilization devices. Their respiratory conditions were almost the same between two immobilization devices as this was performed in a single institution and same physician. Additionally, during the simulation and treatment, the compression devices used are monitored carefully for the position and location of the compression. This study is performed on a single platform and same machine with 4D console with same software for image matching.

We analyzed the interfractional reproducibility for the EBF and the CBP. Intrafractional reproducibility, in contrast, is a substantial concern for SBRT, given the long treatment times. Some studies have investigated the intrafractional errors for the EBF,^18^, ^19^ however, the CBP remains unstudied. One of the limitations of the present study is that no intrafractional data were included for the two devices. Further study of the intrafractional reproducibility should be conducted for the CBP.

The treatment setup time was shorter for the CBP than for the EBF because the CBP does not use a frame and involves fewer points requiring verification by the therapist. This shorter setup time constitutes a clear advantage of the CBP. When the action level was over 3 mm, the differences in incidence rate were 15.0, 20.0, and 17.5% in the LR, AP, and CC directions, respectively, and were larger when compared to the differences seen for an action level of 5 mm. The preparation time was almost the same for both devices with our action level of 5 mm. Institutions where apply action levels of 3 mm will experience a longer preparation time for the CBP than for the EBF because more repeat CBCTs will be needed for the CBP than are needed for an action level of 5 mm.

## 5 CONCLUSION

Our study indicates that the CBP offered shorter setup time, while the EBF required fewer shifts to compensate for interfractional setup error. Satisfactory accuracy for SBRT can be achieved with IGRT in either system, thus making SBRT more adaptable for differences in patient habits and for enhanced comfort.

## ACKNOWLEDGMENTS

This article was supported by the JSPS Core‐to‐Core Program (No.23003) and the JSPS KAKENHI Grant (No.15H04913) and by Health and Labour Sciences Research Grants for Promotion of Cancer Control Programs (H26‐Cancer Policy‐General‐014). This work was performed at Indiana University School of Medicine, Indianapolis under Institutional Review Board exempt study.

## CONFLICT OF INTEREST

None.
